# The role of exosomal molecular cargo in exosome biogenesis and disease diagnosis

**DOI:** 10.3389/fimmu.2024.1417758

**Published:** 2024-06-25

**Authors:** Meijin Liu, Zhenzhen Wen, Tingting Zhang, Linghan Zhang, Xiaoyan Liu, Maoyuan Wang

**Affiliations:** ^1^ Laboratory Medicine, People's Hospital of Ganzhou Economic Development Zone, Ganzhou, China; ^2^ Department of Rehabilitation Medicine, The First Affiliated Hospital of Gannan Medical University, GanZhou, China

**Keywords:** exosomes, molecular cargo, biogenesis, diagnosis, treatment

## Abstract

Exosomes represent a type of extracellular vesicles derived from the endosomal pathway that transport diverse molecular cargoes such as proteins, lipids, and nucleic acids. These cargoes have emerged as crucial elements impacting disease diagnosis, treatment, and prognosis, and are integral to the process of exosome formation. This review delves into the essential molecular cargoes implicated in the phases of exosome production and release. Emphasis is placed on their significance as cancer biomarkers and potential therapeutic targets, accompanied by an exploration of the obstacles and feasible applications linked to these developments.

## 1 Introduction

Extracellular vesicles (EVs) are produced and released extracellularly by almost all cell types and are widely distributed in various body fluids, such as urine, blood, breast milk, saliva, cerebrospinal fluid, amniotic fluid, semen, etc (1–4). Extracellular vesicles can be divided into three main subtypes, microvesicles (MVs), apoptotic vesicles, and exosomes, based on their size, biogenesis pathways, and biological functions (5). Microvesicles, measuring approximately 100–1000 nm in diameter, are directly released from the cell’s plasma membrane (6). Apoptotic bodies, with diameters usually exceeding 1000 nm and resembling platelets in size, are generated through apoptosis and comprise various organelles, intracellular fragments, and cytoplasmic contents (7). Current evidence suggests that exosomes are produced by endosomal pathways and released into extracellular bilayer vesicles, a subgroup of small extracellular vesicles (sEVs) ranging from 30 to 150nm in diameter (8). However, due to the limitations of current isolation methods, most purified vesicles are usually less than 200 nm in diameter. Therefore, the International Society for Extracellular Vesicles has recommended using “extracellular vesicles” as a generic term (8). In this paper, “extracellular vesicles” primarily refers to exosomes unless otherwise specified.

**Figure 1:**
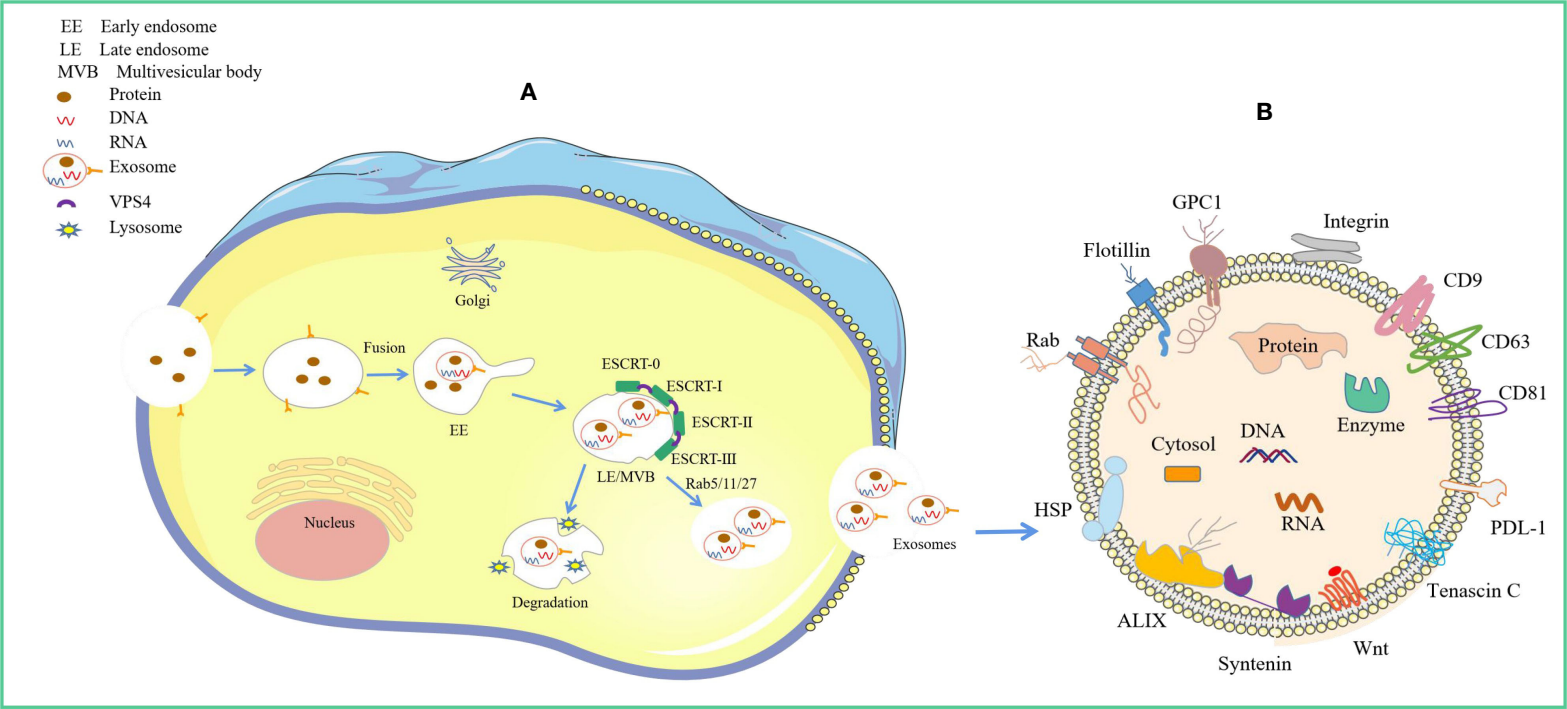
Origin of exosomes and distribution of different exosomal cargo molecules. **(A)** Origin of exosomes. The ESCRT-dependent pathway, the lipid raft and the four-transmembrane protein mechanism play a leading role, and the Rab protein further assists cargo sorting and exosome release. **(B)** Exosome Common Cargo Molecules. Tetraspanin proteins (CD9, CD63, CD81), PD-L1, Integrins; Wnt protein, ALIX, Syntenin, HSPs, tenascin C; GPC1, Rabs, Flotillin, DNA and RNA etc.

The exosome biogenesis and release processes are tightly regulated and achieved by the interplay of different effectors (9) (**Figure 1**). These mainly involve endosomal sorting complex required for transport (ESCRT)-dependent and ESCRT-independent mechanisms (10). It is now understood that exosome biogenesis begins with the endocytosis pathway, where the plasma membrane invaginates to envelop cell membrane proteins and some extracellular components to form early endosomes (11, 12). Subsequently, early endosomes exchange material with other organelles or further mature into late endosomes (LEs), and late endosomal membranes invaginate to form multivesicular bodies (MVBs) containing intraluminal vesicles (ILVs). Then, MVBs bind to lysosomes or autophagosomes for degradation or are transported to the plasma membrane through the cytoskeleton and microtubule network, fusing with the plasma membrane and exocytosed to form exosomes (13–15). Among these, the formation of ILVs, protection of MVBs from degradation, and fusion of MVBs with the plasma membrane are three key aspects in exosome biogenesis and release (16–18). There is a rich literature available substantiating that exosomal cargo molecules (proteins, lipids, and nucleic acids) regulate the whole process (7, 10, 13, 19). However, little is currently known about how these molecular cargo molecules regulate their biogenesis.

Early studies considered exosomes as waste material excreted by cells to maintain homeostasis (20). Subsequent reports have shown that exosomes can transport substances and transmit information between cells, mediating many physiological and pathological processes (21–23). In addition, these vesicles are involved in immune regulation and intercellular communication and mediate the progression of diseases such as cancer, metabolic diseases, degenerative pathologies, and autoimmune conditions (2, 24–27). It is widely believed that the key to the biological function of exosomes lies in their molecular cargo, including proteins, lipids, and nucleic acids. These inclusions not only regulate the process of exosome formation but can also reflect, to some extent, the progression of diseases and act as mediators for treating certain diseases (28). Wei et al. (29) explored novel exosomal biomarkers for early diagnosis and prognosis of colorectal cancer. Plasma samples were assayed and showed significantly higher expression of CD9, CD63, and EpCAM in colorectal cancer (CRC) patients compared to healthy and benign controls (AUC of 0.90 and 0.96, respectively). Li et al. (30) reported that exosomal epinephrine A2 showed superior ability compared to circulating PSA in differentiating between patients with prostate cancer and those with benign prostate cancer, with an AUC of 0.906. The composition of these exosomes may differ significantly between healthy individuals and patients, suggesting certain molecular cargoes in exosomes may be potential disease-specific markers. Harnessing exosomes containing specific molecules to target receptor-diseased cells may enable targeted disease treatment.

This paper explores the role of exosomal cargo molecules in their biogenesis and release, summarizes the key role played by these cargo molecules in disease diagnosis, and concludes with a discussion of the challenges and potential applications of exosome research.

## 2 Molecular cargoes regulating exosome formation

Exosomes are found in biological fluids and act as carriers for the transport of proteins, lipids, nucleic acids, and metabolites into the pericellular environment, playing integral roles in various physiological and pathological processes of the body (31, 32). In addition, several cargo molecules play key roles in exosome biogenesis and release. It has been reported that tetraspanins (e.g., CD9, CD63, CD81, CD82), major histocompatibility complex (MHC) molecules, ESCRT proteins, and Rab proteins are mainly involved (22, 33–35). An increasing body of evidence suggests that lipid components such as ceramides, cholesterol, and phosphatidic acid are also involved in this process (36–39). Molecular cargoes associated with exosome biogenesis and release are summarized in **Table 1**.

**Table 1:** Role of related molecular cargoes in exosome formation.

Molecular cargo type	Processes involved	Role	References
Tetraspanins			
CD63	Exosome biogenesis and release	Alters the physical properties of cell membranes and affects secretion	(40, 41)
CD81	Release of exosomes	Promoting changes in membrane curvature	(42)
CD9	Exosome formation	Increased exosome content loading	(43, 44)
ESCRT			
ESCRT-0	Release of exosomes	Promote deformation of membrane structure and raise ESCRT-1	(45, 46)
ESCRT-1	Release of exosomes	Formation of endosomal membrane structural domains	(47, 48)
ESCRT-2	Release of exosomes	Promote film deformation and shrinkage, raise ESCRT-III	(49–51)
ESCRT-3	Release of exosomes	Powering membrane remodeling through ATP hydrolysis	(52–54)
Rabs			
Rab11, Rab35	Exosome biogenesis and release	Promoting early nuclear endosome or circulating nuclear endosome formation	(55)
Rab27A, Rab27B	Exosome biogenesis and release	Promoting late nuclear endosome formation	(56, 57)
Rab5, Rab7	Release of exosomes	Key molecules in early and late nuclear endosome formation	(58)
Ceramides			
sphingomyelin	Exosome biogenesis and release	Maintaining membrane integrity and stability	(59)
sphingosine 1-phosphate	Release of exosomes	Promoting spontaneous membrane bending and binding to microstructural domains	(60)
Phospholipase			
phospholipase D	Exosome biogenesis and release	Promote germination of intraluminal vesicles	(61, 62)
Cholesterol	Exosome biogenesis and release	Exosome biogenesis and release provide driving forces	(63, 64)

### 2.1 Proteins

#### 2.1.1 Tetraspanins

Tetraspanins are integral membrane proteins with four transmembrane structural domains abundantly found in cell membranes. 33 different tetraspanins have been identified in humans, some of which are involved in exosome membrane formation (65). These proteins may interact laterally with membrane molecules of lipids to form microregions rich in tetraspanin transmembrane proteins (TMEM), which are required to promote vesicle fusion and/or fission (66, 67). Likewise, they can promote membrane bending and binding to actin, potentially via the interaction of tetraspanins with Rho GTPases, affecting actin structure and, consequently, alterations in membrane morphology (68–70). In addition, tetraspanins also aid in the recruitment of exosomal proteins and nucleotides, with CD molecules like CD9, CD63, and CD81 widely recognized as exosomal biomarkers influencing exosome biogenesis and composition (69, 71).

Previous studies have shown that tetraspanins are involved in exosome production in an ESCRT-independent manner, mediated by CD63 and unaffected by ESCRT, ceramide, and ubiquitination pathways (16, 72). CD63 is widely thought to play an important role in exosome formation. In this respect, CD63 knockdown in HEK293 cells was found to reduce exosome production (73). In the brains of patients with Down syndrome (DS), overexpression of CD63 increased exosome release and attenuated endosomal abnormalities (40). Importantly, CD63 may produce large protein structural domains on the inner surface of late endosomes, which then alter the physical properties of the membrane in an invaginated manner, thereby affecting exosome secretion (41). Current evidence suggests that CD81 is the most enriched protein in exosomes (66). Interestingly, the cone-like structure of CD81, which accommodates cholesterol molecules, can promote membrane curvature changes. The aggregation of cone tetrameric proteins induces them to bud inward toward the enriched regional domains, contributing more to exosome formation (42). Other tetraspanins that play a role in exosome biogenesis include CD9, which interacts with the membrane metalloprotease CD10 to enhance the exosome loading of CD10 (43). A recent study by Chairoungdua et al. found that bone marrow dendritic cells (BMDC) from CD9 knockout mice released fewer exosomes than wild-type dendritic cells52, although the underlying mechanism has not been elucidated (44). Besides, the recruitment of major histocompatibility complex class II (MHC-II) to exosomes in antigen-presenting cells (APCs) is independent of MHC-II ubiquitination and depends on CD9-rich microstructural domains (74). Notably, CD9 and CD82 interact with ceramide and secrete β-linked proteins in exosomes, suggesting that tetraspanin-mediated exosome biogenesis is closely related to lipids (44). Future studies should focus on unraveling the specific mechanisms of exosome biogenesis and release for different subsets of tetraspanin molecules, which have potential as biomarkers and can be targeted for therapeutic effects.

#### 2.1.2 Endosomal sorting complex required for transport

In recent years, ESCRT complexes have emerged as key players in exosomal biogenesis by promoting the budding of ILVs and their release into endosomes, thus promoting the formation of multivesicular bodies (75). It has been established that ESCRTs consist of four distinct protein complexes (ESCRT-0, -I, -II, and -III) that bind to vacuolar protein sorting 4 (VPS4), Alix, and VTA1 proteins to promote vesicle budding and cargo sorting in MVBs (76) (**Figure 1**). Protein ubiquitination is a key regulator that mediates the sorting of ESCRT cargo into ILVs (77). The ubiquitinated cargo is recognized and sorted by ESCRT-0 key subunit hepatocyte growth factor-regulated tyrosine kinase substrates into phosphatidylinositol-3-phosphate-rich (PI3P) endosomal compartments (78). Interestingly, ESCRT-0, -I, and -II contain ubiquitin-binding subunits that link ubiquitinated membrane proteins sorting to specific regions of the endosome (45, 49). Importantly, ESCRT-0, -I, -II, -III, and Vps4 are progressively recruited to function on the endosomal surface, thus contributing to endosomal sorting and MVBs generation (46). First, ESCRT-0 is recruited to the endosomal membrane by monoubiquitinated transmembrane proteins to promote the microdomain aggregation of ESCRT-0 in the vacuolar portion of the nuclear endosome while deforming the membrane structure and recruiting ESCRT-1 via the HRS PSAP structural domain interacting with the subunit TSG101 of ESCRT-1 (79). Subsequently, ESCRT-0 and ESCRT-I aggregate cargoes beneath a flat protein lattice coating, forming the substructural domain of the endosomal membrane (47, 48). Given that ESCRT-1 and ESCRT-2 are localized on the late endosomal membrane, ESCRT-III is recruited, leading to membrane deformation and constriction and, ultimately, microdomain budding (49–51). Notably, it is now understood that cytoplasmic proteins are engulfed by ILVs when ESCRT-1 and ESCRT-2 begin to invaginate to form ILVs, although the mechanism of engulfment is unclear (80). Furthermore, ESCRT-III proteins can form filaments that break down ESCRT-III filaments and recycle ESCRT-III complexes via ATP hydrolysis-derived energy in the presence of VPS4 ATPases complexes, which together drive vesicle neck narrowing and ESCRT-mediated membrane remodeling (52–54). Finally, the ESCRT-III complex binds to Vps4 to drive membrane outgrowth away from the cytoplasm while severing the membrane to release ILVs into the lumen of MVBs to form a closed vesicle (81). The above studies overlap in their assertion that ESCRT-III is the main driver of membrane remodeling.

Multiple lines of evidence support the key role of ESCRT mechanisms in exosome formation. For example, deletion of HRS, ESCRT-0 subunit STAM1 (signal transduction adapter molecule), and TSG-101 (tumor susceptibility gene 101) have been reported to reduce exosome release from tumor cells (76, 82). However, the exact mechanism of exosome formation by multiple components and associated proteins of the ESCRT machinery remains unclear. Colombo et al. found that certain components of the ESCRT machinery can selectively act on subpopulations of MVBs and ILVs of exosomes by RNA interference screening. However, the exact mechanism underlying these findings has not been established (76). A recent study by Giordano et al. documented the leptin/leptin receptor/Hsp90 axis as an important regulator of exosome biogenesis in breast cancer cells, mainly due to the interaction of TSG101, a key component of the ESCRT-I fraction, with HSP90 (83). Nonetheless, whether this mechanism is also applicable in other cell types remains to be verified. Although the ESCRT mechanism has been shown to play an important role in ILVs formation, how ESCRT proteins interact to induce ILV outgrowth remains to be further investigated. AlP1/Alix/Vps31, Tsg101/Vps23, and ubiquitinated proteins are widely thought to be required for exosome release from dendritic cells (DCs) (84, 85). In addition, ESCRT components can influence functional changes by controlling exosome formation. For example, the ESCRT-0 component HRS affects the antigen presentation ability of dendritic cells by mediating dendritic cell exosome biogenesis and/or release (50). Latent membrane protein 1 (LMP1) is an Epstein-Barr virus (EBV) oncoprotein that avoids lysosomal degradation. Nkosi et al. found that overexpression of LMP1 increased mRNA and protein expression of CD63, Syntenin-1, HRS, ESCRT-III subunit CHMP6, TSG101, and Alix in nasopharyngeal carcinoma cells, further enhancing EVs production and release. However, knocking down ESCRT-dependent components like Alix and HRS was found to reduce the release of LMP1 EVs, cell proliferation and migration, and tumor growth. This finding suggests that HRS recruits ubiquitinated LMP1 to the endosomal membrane for EVs release or lysosomal degradation (86).

#### 2.1.3 Rab

Rab GTPases represent the most abundant protein family within the Ras superfamily of GTPases that regulate vesicle outgrowth, transport, and fusion processes by recruiting effector proteins (55, 87). Several Rab GTPases have been shown to play important roles in both exosome biogenesis and release, with Rab11 being the first Rab GTPase identified to be involved in exosome release. Rab11 and Rab35 mainly mediate early endonucleosomal or circulating endonucleosomal processes, while RAB27A and RAB27B mainly mediate late endonucleosomal processes (55–57). Importantly, the small GTPases Rab5 and Rab7 are critical for early and late endosomes, respectively, and the class C vacuolar protein sorting/homotypic fusion and protein sorting (VPS/HOPS) complex mediates Rab5 to Rab7 conversion on endosomal membranes (88). It has been reported that Rab11 is required for sustained exosome release within the Drosophila neuromuscular junction (89). Rab35 is present on the surface of oligodendrocytes in a GTP-dependent manner, and inhibition of Rab35 function leads to the accumulation of endosomal vesicles within oligodendrocytes, reducing the docking of MVBs to the plasma membrane and further impeding exosome release (58). Notably, Rab27a can control the docking of MVBs to the plasma membrane in different cell lines to affect exosome release. For example, Rab27a can mediate the docking of MVBs to the plasma membrane in Hela cells, neurons, and podocytes (90, 91). Besides, Rab27a is involved in submembrane actin cytoskeletal rearrangements, and is not required to regulate exosome protein composition (92, 93). It has been shown that Rab27b shares the same function as Rab27a in the endosomal transport of MVBs, i.e., by facilitating the targeting of MVBs to the cell periphery and docking with the plasma membrane. In addition, Rab27a and Rab27b may lead to exosome release via their corresponding effector proteins Slp4 and Slac2b (90).

Although the cell type-dependent regulation of exosome release by Rab proteins has been reported (18), it remains unclear which specific cellular components these proteins act on. Even the role played by the same protein in the release of exosomes differs across cell types. For example, in retinal pigment epithelial cells (RPE1), silencing Rab11 or Rab35 was found to inhibit anthrax toxin exosome release, while silencing Rab27a did not affect exosome release (94). In breast cancer cells, silencing Rab27a resulted in a reduction in exosome release (95). Ostrowski et al. performed RNAi screening of Rab GTPase family members in HeLa cells and found that knocking down Rab27a or Rab27b significantly reduced the amount of exosome release (90). In addition, Rab7 mediates endosomal transport of MVBs to lysosomes and exhibits different roles in cell type-dependent exosome release (58, 96, 97). Thus, the regulation of exosome biogenesis and release by Rab GTPases depends on their unique transport function and the specific cell types in which they operate.

### 2.2 Lipids

Interestingly, lipid molecules in exosomes can promote membrane invagination and induce spontaneous outgrowth of ILVs, mainly due to their structural properties and metabolic characteristics affecting membrane fluidity or curvature (13, 98, 99). At the same time, they may act as signaling molecules to mediate exosome production and release (100). Notably, the distribution of lipids in the two leaflets of the exosome bilayer is asymmetrical, allowing exosomes to present different internal and external signals (101, 102). Given that all EVs are formed by cytoplasmic membrane outgrowth (inward or outward), their lipid composition (ceramides, phospholipids, cholesterol, etc.) may reflect the plasma membrane composition (100, 103). However, the relative amounts of these lipids in the exosome membrane may vary depending on the cell type and function of the exosome.

#### 2.2.1 Ceramides

Ceramide is a tapered lipid whose secretion is dependent on the action of neutral sphingomyelinase (nSMase2). Once ceramide is produced from sphingomyelin (SM), it is readily converted to other bioactive sphingolipids such as sphingosine and sphingosine 1-phosphate (S1P) (59). Importantly, SM contributes to maintaining membrane integrity and stability and is rich in lipid rafts that help regulate cell signaling (104). It has been shown that inhibition of neutral sphingomyelinase (nSMase2) inhibits ILVs outgrowth in MVBs (105). Similarly, inhibition of nSMase2 in T cells and a human embryonic kidney cell line (HEK293) reduced exosome production and release (106, 107). Ceramide also caused spontaneous bending of endosomal membranes and their binding to microdomains, serving as a mechanism for ILVs sprouting (105). Notably, it has been shown that ceramide does not directly affect the maturation and exosome formation of MVBs but may be related to S1P. S1P is a sphingosine phosphorylation product catalyzed by sphingosine kinase (SphK) and is an important factor in the formation and maturation of MVBs (60). S1P receptors on MVBs are critical for sorting cargo into ILVs and S1P receptor activation regulates downstream signaling of Cdc42 and Rac1 activity in Rho family GTPases. The signaling cascade mediated by Gβγ subunits/Rho family GTPases promotes F-actin formation on MVBs, facilitating the sorting of cargo into ILVs. It is highly conceivable that F-actin formation is instrumental in cargo sorting for ILVs. In addition, they found that downregulating specific siRNAs of S1P receptor or SphK2 in HeLa cells resulted in reduced content of ILVs protein cargoes such as CD63, CD81, and flotillin 2 in MVBs (108). However, the potential mechanism of how S1P enters into MVBs to activate the receptor remains to be further elucidated. This also suggests a role for S1P in classifying ILVs as MVBs for exosome release or entry into the lysosomal degradation pathway. Although the interaction between ceramide and S1P has been previously reported to mediate the process of autophagy induction via the mTOR pathway (109), little is currently known about how both regulate the autophagic lysosomal and exosomal release pathways. Furthermore, in a hepatocyte lipotoxicity model, ceramide transport from early endosomes to MVBs mediated by the ceramide transporter protein StAR-associated lipid transfer domain 11 (STARD11) led to ILVs outgrowth and regulated exosome biogenesis (110). In melanoma cells, exosome production is reportedly unaffected by ceramide deficiency (16). Further research is warranted to determine whether ceramide-induced intraluminal vesicle budding in different cell types acts on specific subpopulations of multivesicular bodies. Wei et al. reported that RAB31 could drive the membrane budding of MVBs into the lumen to form ILVs dependent on ceramide in an ESCRT- and tetra-transmembrane protein-independent manner, which was mainly associated with the release of Flotillin protein from lipid raft microdomains (17). This finding suggests that multiple mechanisms may mediate exosome formation.

#### 2.2.2 Phospholipids

Phospholipids also play a significant role in exosome biogenesis and release. Similar to sphingomyelinase (SMase), phospholipase D (PLD) decreases the size of the headgroup in membrane lipids (100). PLD2 in endosomes and exosomes activates phosphatidic acid (PA). This conical phospholipid stimulates ILVs to bud independently of the ESCRT mechanism, mainly due to the negative curvature of the PA head group, which is smaller and may form a cone that facilitates intraluminal bud emergence (111, 112). Importantly, PA can promote membrane rearrangement by generating negative membrane curvature to interact with proteins and by interacting with cargo molecules associated with transporting MVBs and fusion. In addition, PA can facilitate the flipping of endosomal to luminal membranes to aid ILV outgrowth (105, 113, 114). The interaction of PA with syntenin has been reported to trigger the recruitment of syndecan, CD63, and ALIX in the membrane to stimulate the budding process of ILVs (115). In addition, Wu et al. showed that sphingomyelinase interacts with PA to enhance ceramide production and promote ILVs germination in an ESCRT-independent manner (61). Recently, it has been shown that Ral GTPases work synergistically with ARF6 to activate PLD on the membrane of MVBs, which in turn affects PA levels to promote ILV budding (62). Similarly, it has been shown that overexpression of PLD2 leads to increased exosome release, which is mainly associated with PA (116, 117). While studies have demonstrated that phospholipase D regulates various cellular functions through the production of phosphatidic acid, its specific involvement in mediating the formation of intraluminal vesicles and exosome release is still not fully understood, nor is it clear whether PLD primarily acts through PA or in conjunction with other signaling pathways to play a key role in these processes (118, 119).

#### 2.2.3 Cholesterol

Cholesterol plays an important role in exosome biogenesis and release by regulating membrane stability (63, 120). In addition, cholesterol is highly enriched in circulating endosomes (121). Möbius et al. used gas-producing podolysin O to label cholesterol and investigate cholesterol distribution in the endocytic pathway of human B lymphocytes. Cultured B lymphocytes were found to contain both cholesterol-positive and cholesterol-negative MVBs. Interestingly, those cholesterol-rich MVBs could fuse with the cell surface to release exosomes, suggesting that the cholesterol content in MVBs controls exosome release. Besides, some MVBs contained up to 63% endosomal cholesterol (64). Another study revealed that cholesterol might recruit and/or activate different proteins by directly altering the intrinsic fusion properties of the membrane, which in turn facilitates exosome release (122). For example, the ESCRT complex may induce the formation of ordered membrane microstructural domains in a cholesterol-dependent manner, further facilitating ESCRT complex-mediated membrane outgrowth (123). Exosome release is reportedly reduced in bronchial epithelial cell lines (BEAS-2B) due to the cholesterol-lowering effect of statins (124). Similarly, treatment of a hepatocellular carcinoma cell line (Huh-7) with cholesterol decreased the number of MVBs co-localized with lysosomes and stimulated the release of M1-polarized exosomes from THP-1 monocytes (125). Thus, cholesterol content within the endosomal membrane may drive exosome biogenesis and release. The exact mechanism of cholesterol in exosome biogenesis and release is yet to be determined. Moreover, other influencing factors include the parent cell type, the nature of the initial stimulus, and the microenvironment (102).

In summary, exosome biogenesis and release is a complex process that may vary depending on cargo or cellular origin. Furthermore, the process of regulating exosome biogenesis and release involves the coordination of multiple different molecular cargo and signaling mechanisms, with the ESCRT-dependent pathway and lipid raft and four-transmembrane protein mechanisms playing a dominant role, and Rab proteins further aiding cargo sorting and exosome release.

### 2.3 Other regulatory factors

It is well-established that cargo molecules are not the sole regulators of exosome formation. Indeed, the extent of exosome formation and release is influenced by microenvironmental factors, such as hypoxia and eosinophilia. Cargo sorting, transport of MVB, and fusion with the plasma membrane are key steps in exosome release, which may be affected by hypoxia. Cargoes and cargo-sorting machinery are the first regulators of exosome release, and hypoxia may mediate their activity (3). In a recent study, double immunofluorescence analysis confirmed that RAB22A was enriched in microvesicle membranes, suggesting that RAB22A is a carrier of MVs and an elevation in RAB22A expression, dependent on Hypoxia-inducible factors, is crucial for promoting heightened MV formation in hypoxic environments (126). Although microvesicles are different from exosomes, this study provided some insights into the hypoxic regulation of exosomal cargo, which influences exosome release. Exosomal markers, such as tetraspanin membrane proteins (CD81 and CD63) and TSG101, are also good indicators of hypoxic regulation. Many researchers have demonstrated that hypoxia leads to upregulation of tetraspanins. For example, CD63 and GLUT-1 overexpression are hallmarks of hypoxic states and are associated with poor prognosis in patients with gastrointestinal mesenchymal tumors (127). These studies indirectly support the idea that hypoxia may influence cargo loading and subsequent exosome release. In addition, the low pH and acidic microenvironment result from hypoxia, and these features contribute to exosome release and uptake (128). This phenomenon implies that hypoxia can indirectly facilitate exosome release and uptake. Another indirect example is that hypoxia induces exosome release in a calcium-dependent manner involving MCT1 and CD147 (129).

## 3 Exosomal molecular cargoes as diagnostic disease biomarkers

**Figure 2:**
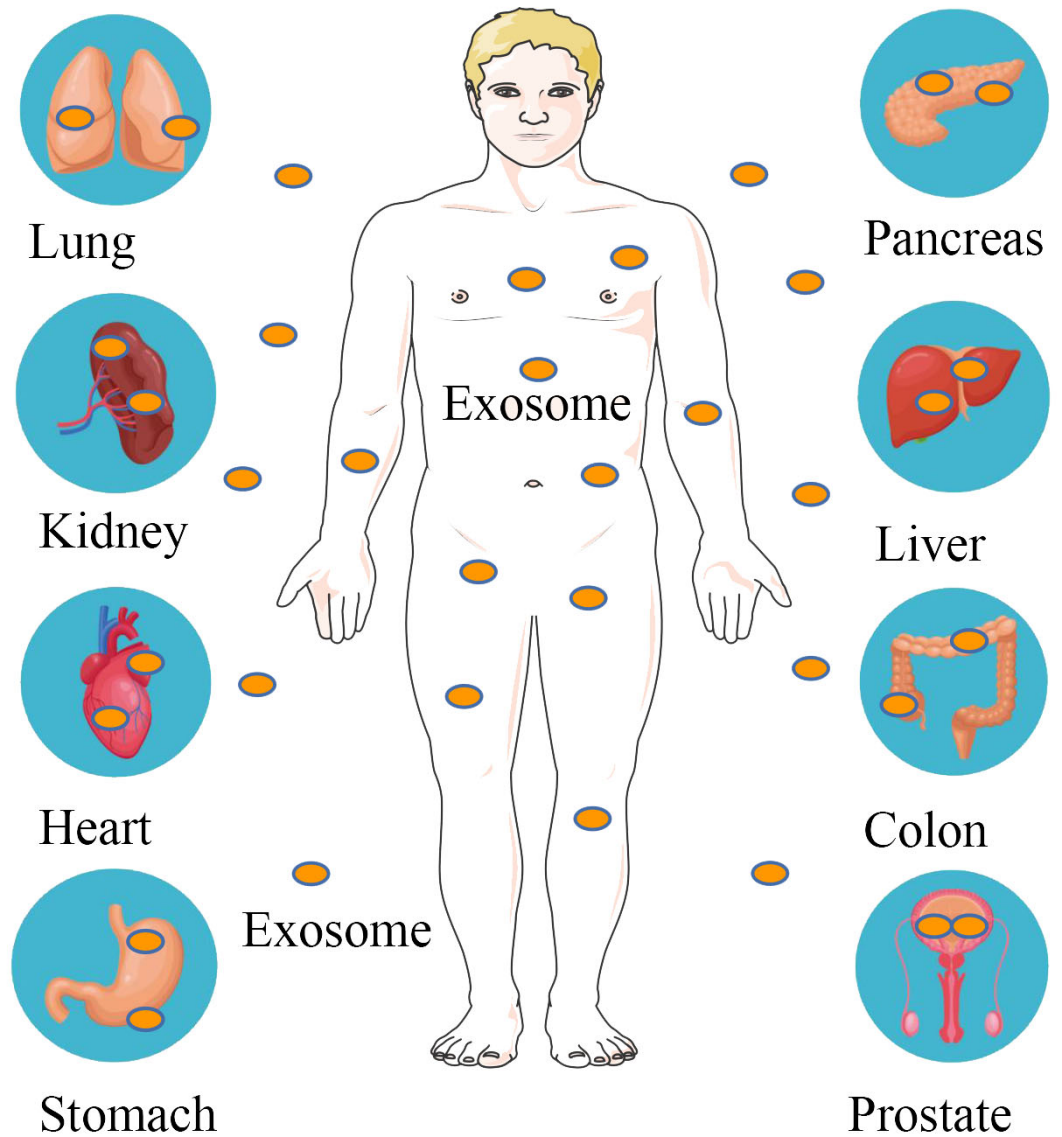
Exosomal molecular cargoes as diagnostic disease biomarkers. Exosomes are distributed in various tissues and organs of the body, including the lungs, kidneys, heart, stomach, pancreas, liver, intestines and prostate. Exosome contents of tumor origin are used to diagnose pancreatic, colorectal, gastric, renal cell, non-small cell lung and prostate cancers.

Exosomal components provide valuable insights into the biological state of a cell and may carry information about the health status of an organ or tissue. An increasing body of evidence suggests that exosomal contents could be harnessed to diagnose various diseases (130–134) (**Figure 2**). **Table 2** summarizes several major classes of molecular cargo that may be biomarkers for common clinical diseases.

**Table 2:** Exosomal molecular cargo as a diagnostic biomarker for disease.

Molecular cargo	Expression	Diseases	Source	Separation method	AUC	Clinical significance	References
Protein							
GPC1	↑	Pancreatic cancer	Serum	Ultracentrifugation	1.0	Early diagnosis and prognostic monitoring	(28)
GPC1	↑	Colorectal cancer	Serum/Tissue	Ultracentrifugation	–	Early diagnosis	(135)
GKN1	↓	Gastric cancer	Serum	Ultracentrifugation	1.0	Early diagnosis and prognostic monitoring	(136)
CP, PODXL	↑	Renal cell carcinoma	Urine	Ultracentrifugation	1.0	Early diagnosis	(137)
Tim-3/Galectin-9	↑	NSCLC	Plasma	Ultracentrifugation	–	Early diagnosis	(138)
mRNA							
CTGF,	↑	Prostate cancer	Serum	Ultracentrifugation	0.86	Early diagnosis and prognostic monitoring	(139)
CAV1	↓	Prostate cancer	Serum	Ultracentrifugation	0.81	Early diagnosis and prognostic monitoring	(139)
MT1-MMP	↑	Gastric cancer	Serum	Sedimentation method	0.78	Diagnosis, treatment and prognosis	(140)
EGFR	↑	Glioblastoma	Serum	Ultracentrifugation	–	Diagnosis	(141)
miRNA							
miR-423-5p	↑	Gastric cancer	Serum	Precipitation method	0.76	Early diagnosis and prognostic monitoring	(142)
miR-19b-3p/miR-106a-5p	↑	Gastric cancer	Serum	Ultracentrifugation	0.82	Early diagnosis and prognostic monitoring	(143)
miR-9-5p	↓	Alzheimer's diagnosis	Serum	Ultracentrifugation	–	Diagnosis	(144)
miR-133a	↑	Coronary artery disease	Serum	Ultracentrifugation	–	Diagnosis	(145)
lncRNA							
UEGC1	↑	Gastric cancer	Plasma	Ultracentrifugation	0.87	Early diagnosis	(146)
HOTTIP	↑	Gastric cancer	Serum	Ultracentrifugation	0.82	Early diagnosis and prognostic monitoring	(147)
GC1	↑	Gastric cancer	Serum	Ultracentrifugation	0.90	Early diagnosis	(148)
CCAT1	↑	Gastric cancer	Serum	Ultracentrifugation	0.89	Early diagnosis	(149)
Lipids							
Phosphatidylserine and lactose ceramide	↑	Prostate cancer	Urine	Ultracentrifugation	0.98	Early diagnosis	(150)
glycerophospholipid		Prostate cancer	Cell supernatant	Ultracentrifugation	–	Diagnosis	(151)
Glycerophospholipids, glycerolipids	↓	Hereditary α-trypsin-like disorders	Urine	Ultracentrifugation	–	Early diagnosis	(152)
Acid sphingomyelinase	↑	Multiple Sclerosis	Cerebrospinal fluid	Ultracentrifugation	0.77	Early diagnosis	(153)

↑, Upregulation; ↓, Downregulation.

### 3.1 Exosomal proteins

Exosomal proteins are widely considered as promising biomarkers for various diseases, given that they encompass a diverse repertoire of protein molecules that mirror the attributes of their originating cells (154). Over the years, exosomal proteins have been identified in different body fluids (e.g., serum, plasma, urine, saliva, and cerebrospinal fluid) exhibiting huge potential as diagnostic tumor biomarkers. For example, Glypican-1 (GPC1), a cell surface proteoglycan belonging to the acetyl heparan sulfate proteoglycan family (155). It has been proposed that GPC1-positive exosomes are highly expressed in the sera of pancreatic cancer patients and the exosomal protein GPC1 (AUC= 1.0) yields significantly better performance than CA19–9 (AUC = 0.739) in differentiating pancreatic cancer patients from healthy controls (28). Importantly, CA19–9 serum levels exhibited limited ability to distinguish between patients with intraductal papillary mucinous neoplasm (PCPL) and healthy controls, whereas GPC1-positive serum exosomes yielded 100% sensitivity and specificity across all stages of pancreatic cancer (e.g., carcinoma *in situ*, stage I, and stages II-IV) (28). Similarly, exosomal protein GPC1 expression was significantly increased in both plasma and tissue specimen types in patients with colorectal cancer; both returned to normal after surgical treatment (135). Another study suggested that downregulated serum levels of exosomal Gastrokine 1 (GKN1) protein may be a valid biomarker for diagnosing gastric cancer (GC) (136).

A recent study by Kugeratski et al. analyzed the exosome proteome in 14 human cell lines and showed that commonly used exosome biomarkers, including CD9, CD63, and CD81, were not commonly present in exosomes from different cell types. However, ALIX, TSG101, and syntenin-1 were present in exosomes from these 14 different cell types, which ruled out the possibility that proteins expressed in very low abundance in these cell lines could be used as exosome markers (132). This finding suggests the heterogeneity in the composition of the four transmembrane proteins across different cell types and indicates a potential mechanism by which ALIX, TSG101, and syntenin-1 are associated with exosome biogenesis. Syntenin-1 was the most abundant protein in the exosome proteome across different cell lines (132), emphasizing the potential of syntenin-1 as a universal exosome biomarker. Notably, Hoshino et al. performed proteomic analysis of extracellular vesicles and granules (EVP) in 426 human samples from tissue explants (TE), plasma, and other body fluids. Their analysis revealed that the levels of CD63 and flotillins exhibit heterogeneity in plasma and tissue-derived EVPs. Moreover, the study found that plasma-derived extracellular vesicles were enriched with immunoglobulins, a protein family that can effectively differentiate between normal samples and those from various cancer types. It was also found that plasma-derived extracellular vesicles were filled with immunoglobulins, which constitute the predominant family of proteins distinguishing normal samples from cancer samples and different cancer types. Additionally, these EVPs were rich in leucine-rich repeat protein 26 (LRRC26), the ATP-dependent translocase ABCB1 (ABCB1), the bile salt export pump (ABCB11), the adhesion G protein-coupled receptor G6 (ADGRG6), bridging granulin-1 (DSC1), desmoglein-1 (DSG1), keratin, type II epidermal Hb1 (KRT81), and fibrinogen-like protein B (PLGLB1). These proteins were only present in plasma-derived EVP from pancreatic cancer (PaCa) patients, but not in tumor tissue (TT) and adjacent normal tissue (AT)-derived EVP or were expressed at extremely low levels, suggesting the potential of these proteins as specific tumor-associated EVP proteins. In addition, they showed that EVP proteins could distinguish cancers during early stage disease for pancreatic (PaCa) and lung (Luca) adenocarcinoma, highlighting their potential as biomarkers for early cancer detection (156).

Raimondo et al. performed proteomic analysis of urinary exosomes from patients with renal cell carcinoma (RCC) and healthy controls. They found that matrix metalloproteinase 9 (MMP9), copper cyanine (Cp), podocalyxin (PC), DKK 4, and carbonic anhydrase IX (CAIX) were significantly enriched in RCC, while Aquaporin-1 (AQP-1), extracellular matrix metalloproteinase inducer (EMMPRIN), enkephalinase (CD10), dipeptidyl peptidase 1 and Syntenin-1 expression were significantly decreased. The diagnostic accuracy of CP and Podocalyxin (PODXL) for renal cell carcinoma was significantly higher, with AUC values equal to 1 (137), suggesting the potential clinical application of CP and PODXL protein molecules for diagnosing renal cell carcinoma. Interestingly, Sun et al. identified members of the membrane-linked protein family (Annexin A1, A2, A3, A5, A6, A11), nitrogen permease regulator 2-like protein (NPRL2), carcinoembryonic antigen-associated cell adhesion molecule 1 (CEACAM1), mucin 1 (MUC1), in salivary exosomes from lung cancer patients by proteomic analysis, in addition to Prominin-1 (PROM1), histone H4 (HIST1H4A) and tumor necrosis factor alpha-inducible protein 3 (TNFAIP3), which have been identified as biomarkers associated with lung cancer (157). Plasma exosomal Tim-3 and Galectin-9 protein molecules exhibited significantly higher levels in patients with non-small cell lung cancer (NSCLC) than in healthy controls. Importantly, exosomal Tim-3 and Galectin-9 expression levels were positively correlated with clinicopathological characteristics such as patient age, tumor size, distant metastasis, and cancer stage. In addition, exosomal Tim-3 was associated with lymph node metastasis. Therefore, exosomal Tim-3 and Galectin-9 may be potential biomarkers for clinical applications in NSCLC (138).

Diabetes mellitus and its associated complications are metabolic diseases with high morbidity that result in poor quality of health and life. A meta-analysis showed that circulating exosomes released by platelets, monocytes, and endothelial cells were significantly increased in diabetic patients; however, exosomes from leukocytes did not differ between diabetic patients and controls (158). In diabetic nephropathy patients, it was observed that the number of urinary podocyte exosomes was significantly higher compared to alterations in other biomarkers, including urinary albumin or renin (which serves as an early indicator of glomerular injury) (159).

These findings suggest that exosomal proteins have huge potential as diagnostic biomarkers. Future studies should focus on expression levels of disease-specific proteins and further refine current techniques to detect exosomal cargo proteins.

### 3.2 Exosomal mRNA

Messenger RNA (mRNA), transcribed from a strand of DNA as a template, represents a class of single-stranded ribonucleic acid that carries genetic information and directs protein synthesis. mRNA is not only an important exosome cargo but also serves as a functional regulator in the process of exosome derivation from cancer cells (160). To compare the performance of circulating exosomal messenger RNA (emRNA) versus tissue mRNA in the differential diagnosis of prostate cancer (PCa), Ji et al. used sequencing and other methods, which demonstrated unique expression patterns between emRNA and tissue mRNA. However, circulating emRNA performed better as a diagnostic biomarker for PCa patients. Receiver operating characteristic curve (ROC) analysis showed that the AUC values for circulating emRNA in screening and diagnosis of PCa patients were 0.948 and 0.851, respectively. Furthermore, six molecules in emRNA, including CDC42, IL32, MAX, NCF2, PDGFA, and SRSF2, were upregulated during the screening and diagnosis of PCa patients compared to healthy controls (161). Similarly, Shephard et al. demonstrated the potential of serum-derived EV-mRNA in the differential diagnosis of prostate cancer. Among them, increased serum-derived EV-mRNA CTGF molecules or decreased EV-mRNA CAV1 molecules were strongly correlated with the rate of disease progression, and the AUC values for CTGF and CAV1 were 0.8600 and 0.8100, respectively. However, serum PSA did not predict disease progression, suggesting that EV-mRNA CTGF and CAV1 are superior to PSA (139). Another study showed that serum exosomal membrane type 1 matrix metalloproteinase (MT1-MMP) mRNA was significantly upregulated in patients with gastric cancer compared with healthy controls and patients with chronic gastritis or atypical hyperplasia, with an AUC value of 0.788, sensitivity of 63.9% and specificity of 87.1%, compared with an AUC value of only 0.655 for serum CEA. In addition, the combination of exosomal (MT1-MMP) mRNA combined with CEA (AUC=0.821) was significantly better than (MT1-MMP) mRNA or CEA alone in identifying GC patients. Importantly, serum exosome (MT1-MMP) mRNA was significantly associated with tumor differentiation, depth of infiltration, lymphatic metastasis, distal metastasis, and TNM stage (140). Recently, it has been suggested that exocrine epidermal growth factor receptor (EGFR) mRNA may be a potential predictor of glioblastoma (141).

### 3.3 Exosomal miRNA

miRNAs represent a class of small endogenous non-coding RNAs consisting of 18–24 nucleotides. It is widely acknowledged that miRNAs delivered to recipient cells can regulate various genes by blocking translation and inducing mRNA degradation (162). Recent studies have revealed that exosomal miRNAs may be potential biomarkers for certain cancers. For instance, Yang et al. found that serum exosomal miR-423–5p levels were highly expressed in patients with gastric cancer, with AUC values of 0.763, 0.596 and 0.607 for exosomal miR-423–5p, serum CEA and CA-199, respectively, suggesting that serum exosomal miR-423–5p may be a potential GC diagnostic biomarker (142). In a recent study, six miRNAs were found to be significantly highly expressed in the serum exosomes of GC patients. The AUC values of these six miRNAs were 0.627 (miR-10b-5p), 0.652 (miR-132–3p), 0.637 (miR-185–5p), 0.683 (miR-195–5p), 0.637 (miR-20a-3p) and 0.652 (miR-296–5p). In addition, the combination of these six miRNAs improved the diagnostic accuracy in GC patients (AUC=0.703) (163). Another study found that serum exosomal miR-19b-3p and miR-106a-5p levels could distinguish GC patients from healthy controls, with AUC values of 0.813 and 0.806, respectively. Similarly, miR-19b-3p combined with miR-106a-5p demonstrated robust diagnostic power (AUC=0.826) (143). The findings of the above studies suggest that exosomal miRNAs represent potential biomarkers for certain diseases.

In recent years, exosomal miRNAs in Alzheimer’s diagnosis (AD) have attracted extensive attention from researchers. A case study found that 71 miRNAs were significantly different in CSF exosomes from normal controls (19 upregulated, 33 downregulated) (164). Another study documented more miRNA abnormalities in AD plasma exosomes (73 upregulated, 342 downregulated) (165). Among the downregulated miRNAs, mir-9–5p exhibited significantly lower levels in the blood of AD patients (144). In the context of coronary artery disease (CAD), exosomal miR-133a was found to be elevated in injured myocardium and dead cardiomyocytes (145). Recently, Liu et al. (166) revealed the therapeutic role of circulating endothelial cell-derived microvesicle miRNAs, especially miR-92a-3p, in regulating the phenotypes of ECs and vascular smooth muscle cells under atherosclerotic conditions, which could be a candidate marker for the predicting prognosis of CAD.

### 3.4 Exosomal lncRNA

In addition to miRNAs, exosomal lncRNAs have attracted interest as potential diagnostic biomarkers. Long-stranded non-coding RNAs (lncRNAs) have been documented in the nucleus or cytoplasm of cells and can interact with DNA, RNA, or proteins (167). Several studies have suggested that exosomal lncRNA may have huge potential as a diagnostic biomarker for cancer. For example, plasma exosome lncUEGC1 was significantly upregulated in patients with stage I or II gastric cancer, and plasma exosome lncUEGC1 (AUC = 0.8760) yielded significantly better performance than serum CEA (AUC = 0.6614) in diagnosing patients with early gastric cancer, indicating that exosomal lncUEGC1 may be a potentially highly sensitive biomarker in diagnosing early GC (146). In addition, serum exosomal long-chain non-coding RNA HOTTIP was found to be a potential diagnostic marker for gastric cancer, with an AUC value of 0.827, higher than CEA, CA19–9, and CA72–4 (AUC values of 0.653, 0.685 and 0.639, respectively). Importantly, HOTTIP expression levels were significantly correlated with the depth of gastric cancer infiltration and TNM stage (147). Another study confirmed that high expression of circulating exosomal long-stranded non-coding RNA-GC1 (lncRNA-GC1) could distinguish patients with early gastric cancer from healthy controls, and ROC curve analysis showed that exosomal lncRNA-GC1 (AUC=0.9033) exhibited superior diagnostic performance than serum CEA, CA72–4 and CA19–9 (AUC values= 0.5987, 0.6816 and 0.6482, respectively) (148). In addition, LINC00152 was significantly elevated in the plasma exosomes of gastric cancer patients. Elevated exosomal LINC00152 has been reported to be a potential diagnostic indicator of gastric cancer with an AUC value of 0.657 (168). Similarly, Xiao et al. demonstrated that the serum EVs of gastric cancer patients exhibited a significantly elevated expression level of lncRNA CCAT1 in comparison to healthy individuals, as well as patients with chronic gastritis or atypical hyperplasia. The AUC for EV lncRNA CCAT1 alone was 0.890, with a sensitivity of 79.6% and specificity of 92.6%. When combined with carcinoembryonic antibody, the AUC value increased to 0.910, with a sensitivity of 80.5% and specificity of 92.6%. In addition, EV lncRNA CCAT1 may promote GC cell proliferation, migration, and invasion through c-Myc or Bmi-1 upregulation (149).

### 3.5 Exosomal lipids

Lipid molecules in exosomes are mainly used to maintain their external morphology. Lipid molecules in EVs have been reported to protect nucleic acid and protein contents from harmful stimuli in the extracellular environment and function as biologically active molecules involved in tumor biological processes (169, 170). It has been suggested that exosome lipid molecules may also serve as potential biomarkers for cancer patients (171–175). In this context, Skotland et al. showed that urine exosomal lipid molecules (such as phosphatidylserine and lactose ceramide) could be used as prostate cancer biomarkers (150). Subsequently, Brzozowski et al. analyzed exosomes released from non-tumorigenic (RWPE1), tumorigenic (NB26), and metastatic (PC-3) prostate cell lines, and found significant differences in the abundance of lipid species across these three different prostate species. The abundance of diacylglycerol (DG) and triacylglycerol (TG) species was reduced in EVs from both NB26 and PC-3 cell lines compared to EVs from the RWPE1 cell line. However, EVs in the NB2 and PC-3 cell lines were enriched in glycerophospholipids compared to EVs in the RWPE1 cell line. In addition, ceramide and SM species did not differ significantly in these three cell lines (151). Exosomal lipid components have been detected in hepatocellular carcinoma (HepG2/C3a and Huh7 cells), melanoma (B16-F10 cells), glioblastoma (U87 cells), and pancreatic cancer (AsPC-1 cells) (176–179). Besides cancer, other diseases can be detected by analyzing exosomal lipids in body fluids. For example, Glover et al. showed reduced levels of exosomal lipid molecules such as glycerophospholipids, glycerolipids, and sterols in the urine of patients with hereditary α-trypsin-like disorders (152). Similarly, overexpression of the exosomal lipid molecule, acidic sphingomyelinase, in the cerebrospinal fluid of patients with multiple sclerosis exhibited a significant correlation with disease severity, thereby opening up new possibilities for diagnosing and managing this condition (153). Among them, phosphatidylcholine (PC), phosphatidylethanolamine (PE), phosphatidylinositol (PI), SM, cholesterol, and ceramide are the common exosomal lipid molecules with significantly different expression in different diseases (101, 125, 180, 181). In addition, EV-derived sphingolipids in tumor cells promote endothelial cell migration and angiogenesis during tumor growth and metastasis (182).

In summary, exosomal nucleic acid, protein, and lipid molecular cargoes have promising applications as cancer diagnostic biomarkers (**Table 2**). While tissue biopsy remains the definitive method for diagnosing tumors, it is an invasive procedure and has certain limitations, especially regarding the amount of tissue that can be sampled. The ideal diagnostic approach should be able to accurately identify tumor-specific biomarkers through non-invasive techniques, ideally at the pre-metastatic stage (183).

Most molecules currently employed as diagnostic tumor markers rely on identifying the presence of marker molecules that are expressed at significantly higher levels than those found in healthy individuals. For instance, PSA is a diagnostic biomarker for prostate cancer, and CEA is associated with gastrointestinal cancers. Usually, these biomarkers show substantial increases, primarily during the advanced stages of tumor progression.

It is important to acknowledge that current diagnostic methods can be influenced by various factors, leading to potential false negatives or false positives. Therefore, there is a need to enhance their sensitivity and specificity. However, exosomes offer promising advantages in this regard. They are present in various body fluids, exhibit stability, and carry molecular cargoes that reflect genetic or signaling alterations in cancer cells. Detecting exosomes as biomarkers at earlier disease stages could potentially improve the accuracy of cancer diagnosis, reducing the reliance on invasive biopsies. The clinical significance of using exosomes as biomarkers for cancer detection cannot be understated and warrants further exploration and research (184, 185).

## 4 Discussion

Exosomes play a vital role in both physiological and pathological processes, as they are released by diverse cell types and can be found in nearly all body fluids. Acting as messengers in cell-to-cell communication, exosomes play a vital role in normal physiological and pathological processes. Exosome biogenesis and release is a complex process in which molecular cargoes play an important role. It is now understood that the ESCRT-dependent pathway and the lipid raft and tetra-transmembrane protein mechanisms play a dominant role, with Rab proteins further aiding cargo sorting and exosome release. Likewise, lipid components such as ceramide, cholesterol, and phosphatidic acid are involved in the process. There is growing evidence supporting the potential advantages of using exosomal molecular cargoes in disease diagnosis. Exosomal contents can directly mirror the status of secretory cells. Their small size enables them to traverse the body’s tissue barriers and they are prevalent in various body fluids, rendering them easily detectable in clinical settings. Moreover, exosomes feature a lipid bilayer structure that shields their contents from enzymatic degradation in the bloodstream. There is growing evidence that the tumor microenvironment may influence exosome content and alter organismal status. It has been suggested that in the presence of hyperglycemia, exosomal macrophages could participate in the development of muscle insulin-resistance and chronic inflammation (186). This suggests that the microenvironment may be related to exosome biogenesis.

Although tissue biopsy remains the gold standard for cancer diagnosis, its limitation has been gradually revealed in the era of precision cancer therapy. Thereinto, exosomes show the superiority of high sensitivity, specificity and stability compared to other biological components of liquid biopsy like CTCs and ctDNA. For the past few years, a growing number of studies report that exosomal nucleic acid and protein play a pivotal role in tumorigenesis and tumor progression, which indicate that they can serve as a diagnostic or prognostic biomarker. Nonetheless, the studies concerning exosomal lipids and metabolites as diagnostic or prognostic markers are insufficient. Though metabolomic or lipidomic profiling of exosomes in some cancer types including prostate cancer and pancreatic cancer has been conducted the performance of identified exosomal metabolites or lipids in clinical diagnosis and prognosis prediction remains to be further evaluated in a larger sample size meanwhile their roles in tumorigenesis and tumor progression should be explored.

Indeed, obtaining pure and homogeneous exosomes for comprehensive analysis remains a challenge, thus limiting the clinical application of exosomal cargo molecules. The most difficult aspect of exosome research is their isolation and acquisition. Addressing this challenge will significantly advance exosome research. Fortunately, an exciting method has been developed to directly capture exosomes from plasma, serum, or urine. This approach utilizes a range of exosomal membrane proteins, making it possible to isolate exosomes with minimal sample preparation, eliminating the need for vesicle separation (187). Therefore, future strategies for the study of exosome contents can be divided into two categories: (1) isolation and purification of exosome contents for further study of exosomes. This approach is limited by the difficulty of obtaining high-purity exosomes with current technology; (2) immunocapture methods can capture exosomes directly in body fluids and be used for analysis of the contents. However, this method requires the targeting of specific specific antibodies, and the sensitivity of the antibodies used and possible inhibitors of the reaction can affect the accuracy of the results. In addition, exosomes found in body fluids display a remarkable degree of heterogeneity, underscoring the significance of tracking their source. Variations in the expression of specific contents in these exosomes may provide insights into their origins. In the future, it would be valuable to explore the possibility of categorizing exosomes in body fluids based on their content, akin to the classification of blood cells. If this can be achieved, these exosomes may serve as a valuable tool for the diagnosis and treatment of diseases.

## 5 Conclusion

In summary, exosomal cargo molecules play an important role in their biological origin. In addition, exosomes present an exciting research avenue with potential applications in disease prevention, diagnosis, and therapeutic approaches.

## Author contributions

ML: Writing – original draft. ZW: Writing – review & editing. TZ: Investigation, Writing – review & editing. LZ: Data curation, Writing – review & editing. XL: Formal analysis, Writing – review & editing. MW: Project administration, Validation, Writing – review & editing.
